# Perceptions of and Opinions on a Computerized Behavioral Activation Program for the Treatment of Depression in Young People: Thematic Analysis

**DOI:** 10.2196/19743

**Published:** 2021-04-13

**Authors:** Lucy Tindall, Paul Toner, Antonina Mikocka-Walus, Barry Wright

**Affiliations:** 1 Department of Health Sciences University of York York United Kingdom; 2 School of Psychology Queen's University Belfast Belfast United Kingdom; 3 School of Psychology Deakin University Victoria Australia

**Keywords:** depression, qualitative, thematic analysis, young people, health care professionals, computerized therapies

## Abstract

**Background:**

Depression is one of the leading causes of illness and disability in young people, with approximately 20% having experienced a depressive episode by the age of 18 years. Behavioral activation (BA), a National Institute for Health and Care Excellence–recommended treatment for adults with depression, has shown preliminary support for its use with young people. BA may have the potential to be adapted and delivered in a computerized format to address the barriers often associated with young people accessing support. Despite the benefits of adopting computerized therapy delivery, the limited effectiveness of some programs has been attributed to a failure to tailor interventions to patients and practices. Therefore, while developing new treatments, it is important that target users be involved in the intervention design.

**Objective:**

This qualitative study aims to explore the views and preferences of young people and health care professionals regarding the development of a new computerized BA therapy for young people with low mood or depression, to ensure that the therapy was suitable for the target user.

**Methods:**

Semistructured focus groups and individual interviews were conducted with young people (those with experience in accessing support and those without) and health care professionals regarding the development of a new computerized BA therapy for young people with low mood or depression. The data were analyzed using thematic analysis.

**Results:**

A total of 27 individuals, comprising both health care professionals and young people, participated in this study. Vital information pertaining to the important components of a new therapy, including its presentation, delivery, and content, was collected.

**Conclusions:**

Variations in perspectives highlighted the need to adopt a systemic approach in therapy development by considering the opinions of young people with and without experience in accessing mental health support and health care professionals.

## Introduction

### Background

Approximately 20% of young people experience at least one depressive episode by the age of 18 years [1], making depression one of the leading causes of illness and disability in this group [2]. Despite this, help-seeking is limited only to approximately 35% of young people [1] because of factors including stigma [3,4], negative attitudes about help-seeking [4], accessibility [3], and reluctance to engage one-to-one with a therapist [5]. Much attention has been placed on computerized delivery of therapy, in particular cognitive behavioral therapy (CBT) [6-8], which removes some of these barriers. One alternative to CBT is behavioral activation (BA), which encourages increased engagement in adaptive activities and decreased engagement in activities that might maintain or increase depression risk [9]. Owing to its empirical support [10-13], BA is a National Institute for Health and Care Excellence–recommended treatment for adults with depression [14]. Given its demonstrated effectiveness and simple delivery, BA may have the potential to be adapted for use with young people and delivered in a computerized format.

Despite the evident benefits of adopting a computerized approach to therapy delivery, including increasing therapy accessibility, availability [15], and anonymity [16]; reducing stigmatization [17]; and providing those reluctant to engage one-to-one with a therapist with access to care [18], the limited effectiveness of some programs has been attributed to a failure to tailor interventions to patients and practices [19,20]. Therefore, in the development of new treatments, it is important that those for whom an intervention is being designed are involved in its development [21,22], and qualitative research is particularly useful in this context.

When designing a computerized BA program, those deemed the best to inform the development include not only those who may use such a program but also those involved in treatment delivery. Research suggests that one important process that may enhance treatment effectiveness is the incorporation of young people’s views in treatment design to ensure that the developmental needs of the users are considered [23]. However, some depression and anxiety treatments for young people are simply downward extensions of adult therapies, as reflected in both their design and delivery [24,25]. Therefore, the needs and capacities of young people are not always considered when such information is being collected from adults, including parents and pediatric health care professionals [26]. This omission to collect information firsthand may result in the misinterpretation of young peoples’ needs and a focus on the needs of adult representatives instead [27], highlighting the need to include young people when developing interventions designed for them.

In research that has incorporated the views of young people, differences have been evident between their perceptions and those of health care providers. When reviewing research related to the experiences of care systems, health care professionals’ priorities contrasted with the key points highlighted by young people [27]. Furthermore, an examination of illness experiences through interviews with 7 children who had been hospitalized showed that illnesses had different meanings for young people compared with those for professional caregivers [28]. Here, both the illnesses and the mechanisms of recovery were seen in very different ways. Although young people had subjective and personal knowledge of illnesses derived from their own experiences, health care professionals based their knowledge on easily measured objective symptoms. Therefore, there is a need to encapsulate the perspectives of both health care professionals and young people in the development of a computerized BA program.

### Objectives

This qualitative study explores the views and preferences of young people and health care professionals regarding the development of a new computerized BA therapy for young people with low mood or depression. The information gathered about the treatment components that such a therapy should include and how it should be presented and delivered inform the development of the new treatment.

## Methods

### Overview

This research adopted an exploratory, qualitative design using a critical realist approach [29] and was conducted within 4 UK National Health Service (NHS) trusts. Ethical approval was granted by the Health Research Authority (reference: 16/EM/0420) and the Department of Health Sciences Research Ethics Committee at the University of York.

### Participants, Sampling, and Recruitment

In total, 3 groups of individuals participated: young people from a community sample, young people from a service user sample, and health care professionals with experience of working with young people with low mood or depression. Participants were identified using a combination of purposive and snowball sampling approaches.

#### Young People

As many young people experiencing a depressive disorder do not seek help [1], a community sample of young people from a local school within the remits of one of the participating NHS trusts was invited to a focus group. A purposive sampling approach was used, stratifying those identified by age and gender to increase the representativeness of the participants. In addition, a sample of young people receiving support for depression within 3 local child and adolescent mental health services (CAMHS) were identified by health care professionals and invited to attend face-to-face, semistructured interviews with the researcher. Recruiting young people from both a community and a health care sample allowed maximum variation in the views collected. The recruited participants were males and females aged between 11 and 16 years. This ensured that those recruited represented a sample similar to the target group that the eventual program was to be designed for.

All prospective participants received information about the study, with those expressing interest providing written assent alongside consent from a parent or guardian.

#### Health Care Professionals

A sample of health care professionals with experience of working with young people with low mood or depression from 2 CAMHS was invited to a focus group or an individual interview if more convenient. Anyone expressing interest provided written consent for participation.

### Data Collection

All focus groups and interviews were conducted by the lead researcher within the schools and CAMHS sites where recruitment occurred. Before all interviews and focus groups, attendees completed a short questionnaire—young people were asked to supply basic demographic information and health care professionals provided information about their clinical practice.

The interviews and focus groups closely followed 1 of 2 interview schedules (one for young people and one for health care professionals; Multimedia Appendix 1), comprising semistructured, open-ended questions. The interview schedules for young people were based on collecting information pertaining to 4 different domains: previous experiences of web-based help and opinions regarding the new program development, the content and treatment-related activities to be considered for inclusion in the new program, delivery-specific information (eg, the number and structure of sessions, access, and program presentation), and parental involvement. Although a similar structure was adopted in the interview schedules for health care professionals, they were also asked questions related to the new program’s positioning in the care pathway and about perceived support requirements for program completion. With permission, all interviews and focus groups were audio recorded digitally and transcribed verbatim.

During the focus groups and interviews, participants completed 2 bespoke activities specifically developed for the research. The first activity, which was completed by all, was used to elicit further information regarding the content to be considered for inclusion in the new program, with individuals asked to indicate what they deemed to be important from a list of potential options (eg, activity scheduling, activity monitoring, and relapse prevention). Definitions of terms were provided as part of this activity to aid in participants’ understanding. This list was generated from a review of the treatment components often included in BA [30] and the findings from a previous systematic review and meta-analysis of BA used in young people [31]. The second activity, which was completed by young people only, was used as an additional tool to collect delivery-specific information. This involved young people ranking 10 factors that they felt would make the program more attractive to young people (interactivity, colorfulness, etc) in order of importance. The activities allowed for methodological triangulation [32], made participation more enjoyable, and allowed individuals to provide information in various ways.

### Data Analysis

Data were analyzed using thematic analysis, which was selected to provide rich and detailed insights about a given topic [33]. The analysis was both inductive and deductive, with themes or subthemes generated from both the raw data provided by participants and from theory and previous research. Thematic analysis comprises 6 phases: familiarization with the data, generating initial codes, searching for themes, reviewing themes, defining and naming themes, and reporting production [33]. By closely following these phases, themes were identified from what participants perceived to be the important components of a computerized BA program for use with young people experiencing low mood or depression. All analyses were completed by hand using Microsoft Word, and no specific qualitative software packages were used.

## Results

### Overview

**Figure 1:**
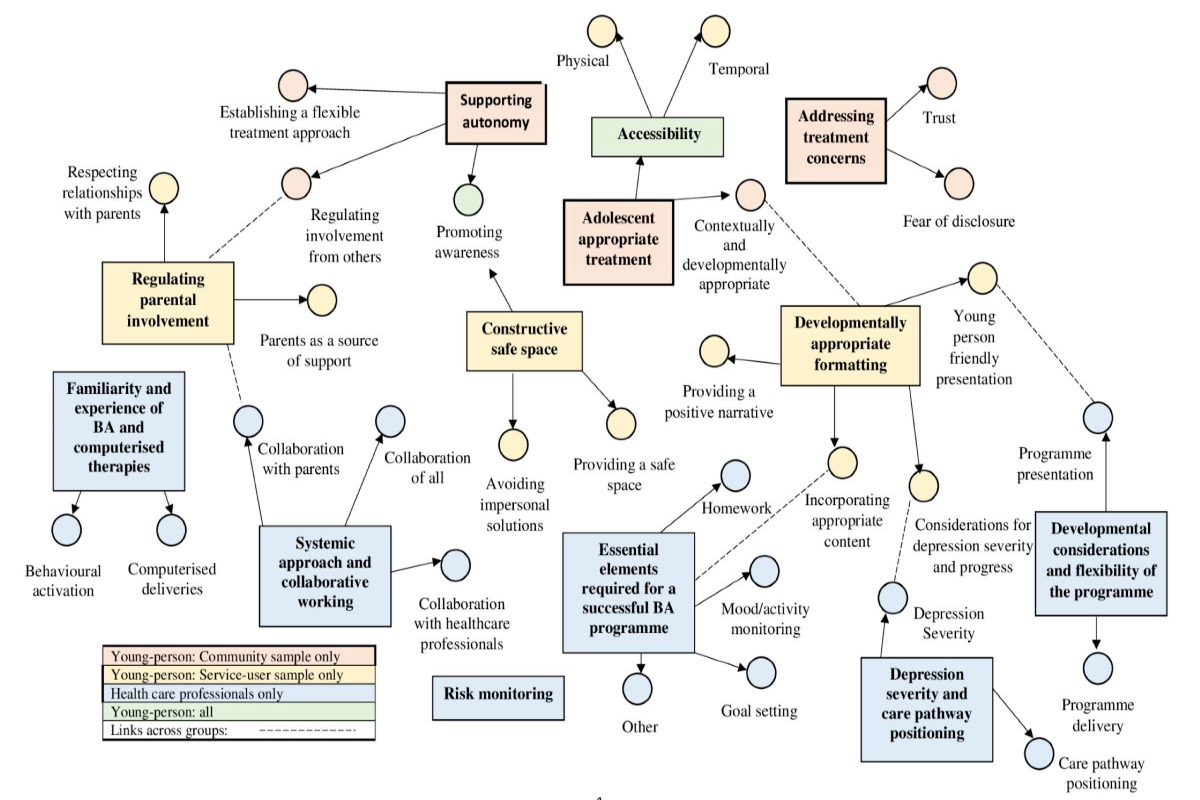
Thematic map of young person participants and health care professionals’ perspectives. BA: behavioral activation.

In total, 27 individuals comprising young people from a community sample, young people from a service user sample, and health care professionals attended interviews and focus groups with the lead researcher. Participant demographic information is presented in Table 1, whereas the themes identified in the thematic analysis and their interrelations across groups are presented in Figure 1.

**Table 1:** Participant sample characteristics (N=27).

Participant characteristics				Young person: community sample (n=9)		Young person: service use sample (n=9)		Health care professional sample (n=9)
**Gender, n (%)**								
	Male			5 (55)		1 (11)		2 (22)
	Female			4 (45)		8 (89)		7 (78)
**Ethnicity, n (%)**								
	White-British			8 (88)		9 (100)		8 (88)
	White-Irish			N/A^a^		N/A		1 (11)
	Black-African			1 (11)		N/A		N/A
**Young-person participants only**								
	Age (years), mean (SD; range)			13.8 (0.83; 13-15)		15.2 (1.09; 13-16)		N/A
	**Experience of low mood or depression, n (%)**							
		Yes	2 (22)		9 (100)		N/A	
		No	7 (78)		N/A		N/A	
**Health care professional participants only**								
	**Age group (years), n (%)**							
		18-30	N/A		N/A		2 (22)	
		31-50	N/A		N/A		2 (22)	
		51-70	N/A		N/A		5 (56)	
	**Highest education level, n (%)**							
		Secondary education	N/A		N/A		1 (11)	
		Trade or technical or vocational	N/A		N/A		1 (11)	
		Professional degree	N/A		N/A		1 (11)	
		Bachelor’s degree	N/A		N/A		1 (11)	
		Master’s degree	N/A		N/A		3 (34)	
		Doctorate	N/A		N/A		2 (22)	
	**Job role, n (%)**							
		Nurse	N/A		N/A		4 (45)	
		Clinical psychologist	N/A		N/A		2 (22)	
		Student mental health worker	N/A		N/A		1 (11)	
		Support worker	N/A		N/A		1 (11)	
		Art therapist	N/A		N/A		1 (11)	
	**Practice years, n (%)**							
		0-5	N/A		N/A		3 (33)	
		11-15	N/A		N/A		1 (11)	
		20+	N/A		N/A		5 (56)	
	**Computer proficiency, n (%)**							
		Fair	N/A		N/A		2 (22)	
		Good	N/A		N/A		4 (45)	
		Very good	N/A		N/A		3 (33)	

^a^N/A: not applicable.

### Young People: Community Sample

In total, 3 main themes were identified: supporting autonomy, addressing treatment concerns, and adolescent-appropriate treatment.

#### Supporting Autonomy

Most young people in the community sample were unsure of how to access support if required, with only 2 having ever received any support—one for bullying support and another for dealing with their parents’ divorce. Most assumed that, if they needed any support, their parents would access it on their behalf. None of the users had ever accessed any web-based support, with the majority unaware of its existence, thus highlighting the need for increased awareness of its availability:

I think that’s why a lot of kids nowadays are feeling more pressured into committing suicide and things like that because it’s like “oh I can’t get any help” because they don’t know what to do.

Male, community, aged 13 years

Young people wanted the opportunity for autonomy if using the new program, highlighting the need for flexibility and wanting to make treatment decisions. Therefore, they wanted much of the program to be optional, with users choosing what to view and when. Examples included deciding whether to disclose information about experiences of low mood or depression, selecting which activities to participate in, and deciding whether to complete additional follow-up sessions if available. They also wanted to decide how long they spent on sessions and the frequency of logging on. However, if flexibility with session length was not possible, approximately 30 minutes per session was regarded as reasonable.

Wanting to be independent and able to make treatment-related decisions, many young people did not want parents to be involved, reporting that such involvement could be detrimental to improving mood. Particular concerns included feeling that parents would not understand young person–specific issues:

I’m not being disrespectful to them, but they’ll think they know everything about kids and stuff, but they really like, they don’t.

Male, community, aged 13 years

Friends were suggested as an alternative support to parents, but shortcomings of this approach were identified, including concerns that this could place great pressure on friends who might worry too much, who may not take things seriously, or who could be patronizing. Therefore, if any risk was presumed, young people felt that parents needed to be informed. Consequently, they contended that the new program should include some information for parents but that this should be both minimal and optional, so young people could retain autonomy.

#### Addressing Treatment Concerns

Concerns were raised about how young people would be treated if they accessed support. In particular, they worried that information disclosed would be reported back to parents who may then treat their child differently and place restrictions on them that could worsen their low mood:

...your mum and dad would be like “right I’m not letting you out the house, I’m not letting you do this, I’m not letting you do that” because that kind of puts you down more.

Male, community, aged 13 years

Such concerns were not limited to parents, with some worried about what would happen if they were to report low mood to health care professionals, particularly general practitioners. For most participants, the prospect of computerized therapy delivery allayed some of these concerns. However, for one individual, even accessing therapy in a computerized format could lead to others finding out about their low mood and treating them differently. Specifically, this individual was worried that if they felt particularly low and accessed computerized therapy, people might arrive at their house to tell them “don’t kill yourself.”

Young people wanted assurance that, if accessing computerized therapy, confidentiality would be maintained. They suggested having good security with individual usernames and passwords required for access, incorporating automatic log-offs after short periods of inactivity, and ensuring no personal information requests were made. They also wanted the program’s name to be discrete and not indicative of its purpose.

A consensus emerged that young people would only disclose information about how they were feeling to another person if trust was both established and retained. One young person had concerns about trust if therapy was delivered in a computerized format, stating that “anyone can set up a website.” This was different for others, with one individual highlighting that if therapy was set up by a reputable source, trust could be gained. Second, the anonymity of a computerized approach meant that establishing trust was not an issue.

#### Adolescent-Appropriate Treatment

The community sample felt that the adolescent period was where computerized therapy could be of most importance, particularly for those already experiencing low mood or depression. They outlined specific stressors, including the transition to secondary school, changes to curriculum and workload, and working toward and completing the General Certificate of Secondary Education.

Overall, they felt that 2 or 3 different presentations were needed to suit the target age range, with developmental level guiding the program presentation and chronological age guiding the content (ie, to ensure the therapy did not place greater pressure on young people at already stressful times, such as during examination periods):

A low level sixteen-year-old might have the same ability as a high level twelve-year-old.

Female, community, aged 14 years

The community sample identified communication, goal setting, activity scheduling, and identifying barriers as the most important components for inclusion. In particular, they felt that communication would help users to develop their social skills and described how the inclusion of links to clubs would aid activity scheduling. Additional content for consideration included games or puzzles, if these were not *childish*, and videos, if these were nonpatronizing, modern, showed depression in a “serious way,” and included actors that users could identify with. Several young people wanted homework to be included but only if it were called something else and minimal, thus considering other demands on users’ time. If information about depression was included, young people wanted this to incorporate depression statistics, information about coping strategies, and current treatments. Advice from health care professionals was requested as part of any problem solving. Although recording activities and moods was the least favored component for inclusion (with some participants concerned that this could lead to information disclosure), others wanted this to be included to allow connections to be made between periods and moods.

The colorfulness of the new program, including information about depression and having additional follow-up sessions, was regarded as the most important component in making the new program attractive to users, whereas having printable handouts and homework activities was considered less important. Participants also wanted the program to be sensitive, fun, and relaxed and to provide adequate information—not too much so that users would be overwhelmed or too little so that they would not take it seriously.

Concerns were expressed that not all young people would be able to access a computerized program with proposals made to place it in accessible locations (eg, general practice [GP] surgeries) to ensure equal access. Furthermore, young people wanted the program to be available at all times and therefore accessible not only when most convenient but also when most required. Consequently, young people wanted the program content to be downloadable to ensure it could be viewed even if an internet connection was unavailable. Program access also had to be simple, with no requirements for young people to trawl through terms or conditions or have to “sign up now,” which they described would dissuade them from using the program.

### Young People: Service User Sample

A total of 4 main themes were identified: constructive safe space, developmentally appropriate formatting, accessibility, and regulation of parental involvement.

#### Constructive Safe Space

In total, 4 service users had accessed web-based support, searching the internet and browsing websites, generally when they felt particularly low and when alternative support was unavailable. Although 2 users had found web-based help useful, shortcomings of the approach were described, including a lack of personal rapport, automated responses described as “robotic,” and a feeling that information was more directed at parents:

I found the [name of website] one to be a lot of phone numbers and it was a lot more directed towards parents than the child.

Female, service user, aged 16 years

All the service users had received face-to-face support for low mood or depression, with the majority describing how hard they had found this, particularly talking with another person about their feelings. Similar to the community sample, their main concern related to how they would be perceived, with several reporting feelings of embarrassment or worry that others would not understand. Consequently, they felt that a computerized treatment approach addressed some of these difficulties, providing young people with anonymity that allowed them to talk freely without judgment, in a format that they were both familiar with and comfortable using:

...our safe space is talking through a phone, through a screen because that seems easier than talking to people.

Female, service user, aged 15 years

Other perceived benefits included enabling young people to access support without having to ask for it and the opportunity to receive it within their own surroundings where they felt most comfortable.

None of the service users had ever had any web-based therapies recommended to them or been directed to it as part of therapy. They were therefore keen to ensure that young people were aware of its availability and suggested advertising the new program in health care settings and schools and via social media.

#### Developmentally Appropriate Formatting

The consensus was that 2 versions of the new program were needed. A simpler version was proposed for younger users aged 11-13 years, with more in-depth information presented for older individuals aged 14-16 years.

The service users wanted the new program to be fun, relaxed, and nonpatronizing while being simple, so that young people would not disengage:

If an eleven-year-old who is anxious, low mood, stressing out about themselves is answering questions or reading words that they don’t understand, it is only going to make it worse for them.

Female, service user, aged 15 years

Furthermore, it was highlighted that many young people, especially those with low mood or depression, tend to procrastinate; therefore, the simpler and more focused the new program, the more likely young people would be to use it.

The activities considered most important for inclusion included learning about coping, playing games or puzzles, practicing new skills, and having information about relapse prevention. Several service users had previously used mood monitoring, finding it useful in allowing them to monitor their feelings over time; therefore, this was suggested for inclusion by some users. Additional components identified included information about exercise and other conditions (eg, eating disorders), a chat option so that users could communicate with similar others, useful telephone numbers, and satisfying videos (videos designed to reduce anxiety and aid relaxation). Homework was considered the least important for inclusion, with the requisite that, if included, it had to be minimal to increase adherence and avoid people *getting bored*.

Some users wanted notifications included to remind users to complete sessions and the option to print content. However, these were not favored by all. Some users felt that as long as program content was downloadable, printable content was not required, and it would be easier to keep all information in one place, that is, on the computer. Although some users could see the benefits of notifications, they felt that these needed to be optional, with users controlling how and when to receive them.

To make the program more attractive to users, its interactivity and its availability for completion were considered the most important components, whereas homework and having printable handouts were considered less important.

Although the majority of service users wanted information about depression presented in the new program, several concerns were raised. Some worried that information of this type could make some people feel worse, and therefore, this needed to be optional. Similar concerns were also discussed regarding the recording of activities and moods and watching videos:

I know the videos are to show that people have overcome it, but the only other thing I would say is that I remember looking at people and stuff like that and then I’d feel bad because I’d feel like “well they’ve got it worse than me, so I’m just being dramatic for no reason.”

Female, service user, aged 16 years

The service users felt that the program length—both its session number and the time needed to complete them—required flexibility. Although they had made suggestions about this, they felt that more or less time could be spent on the program relative to how users were feeling and how they were progressing through it. Therefore, anyone noticing positive changes and feeling better would cease program use, whereas those who did not would use it for longer. They also proposed that anyone experiencing a relapse should be offered a follow-up session or be able to repeat the new program in its entirety.

#### Accessibility

The service users felt that through computerized therapy delivery, more young people would be aware of and more likely to access support. They reported that “nowadays everyone has access to the internet” and therefore wanted the new program to be widely available and not restricted to specific locations (eg, GP surgeries or CAMHS sites). This was deemed especially important for those who might struggle to access face-to-face support (eg, those who are too anxious or have transportation issues) and would help to address lengthy waiting lists in CAMHS:

The process of getting into CAMHS is like a bit of a pain with waiting lists and then maybe not having enough sessions and getting discharged before you’re ready and stuff because the fact that there are so many people that need to use the service, but if it’s internet, like, as many people can use it as they need I’m guessing.

Female, service user, aged 15 years

Although the service users felt that internet access would not be a problem for most young people, they did however recommend that all program content should be downloadable so that individuals could ensure program access even without an internet connection.

Although some users felt that it should be available to all, others felt that, to monitor risk, only those given access by a clinician should be able to use it. One individual suggested that clinicians could send program log-in details to anyone they deemed suitable or their parents.

Although variation in the number of sessions was proposed, the consensus was that this should be dependent on the level of support required. Furthermore, some form of follow-up, to review progress and ensure therapy did not simply stop, was regarded as important. There was variation in how this could be incorporated, with some users suggesting a single session after therapy completion to be sufficient and others wanting monthly follow-ups for up to 6 months. One individual suggested making follow-ups optional and aimed at those who might not have processed all of the program content.

The service users felt that the program length needed to consider the attention spans of young people, with most deeming between 30 and 45 minutes per session as sufficient. They felt that young people might *lose focus* if sessions were too long and proposed that larger topics could be covered in multiple sessions to keep session lengths shorter.

Some users felt that weekly sessions with time to practice skills would be appropriate, with concern that users may forget what they had done in a session if longer than a week was left until the next. Others wanted a higher frequency of sessions, with both completing 2 sessions per week and completing sessions on alternate days. It was also suggested that program content should remain accessible following completion so that it could be repeated if needed.

#### Regulating Parental Involvement

Several service users felt that there should be no parental involvement in the new program with concerns that those not having a close relationship with their parents would not engage with a parent present. Some felt that parental involvement should be decided by the user, whereas one service user felt that parents should be informed if their child was using the program simply to show them that they were using the computer in an appropriate way.

In contrast, parental involvement was regarded positively by some users who felt that the program could be completed more successfully if parents were involved. These individuals described how this could result in parents gaining a better understanding of how their child was feeling and how parents could learn techniques to be able to provide more suitable support:

If parents have techniques for the child, they could then show them techniques, you know that could help them, or maybe learn not to maybe act a certain way with them and stuff.

Male, service user, aged 16 years

Handouts providing information about low mood or depression and coping techniques were suggested as a way of implementing parental involvement. There were also suggestions about including parents in some sessions with variations, including young people completing sessions together with parents, having parent-only and young person-only sessions completed separately, or combining these approaches with parents attending sessions at various points.

### Health Care Professionals

In total, 6 main themes were identified: familiarity and experience of BA and computerized therapies, essential elements required for a successful BA program, developmental considerations and flexibility of the program, depression severity and care pathway positioning, risk monitoring and systemic approach, and collaborative working.

#### Familiarity and Experience of BA and Computerized Therapies

Most health care professionals were familiar with the components of BA, having used some of them—mainly mood and activity monitoring—in therapy. However, most health care professionals, unaware of the term, did not refer to this as BA. Those who were familiar with BA described how their knowledge of the treatment was derived from an adult model. Therefore, if they had applied BA principles to young people, this was through them adapting their knowledge of BA with adults and creating a bespoke treatment approach.

All health care professionals were familiar with, and valued, the increased use of computerized therapies, with some having referred young people to web-based resources while awaiting face-to-face therapy. Despite this, limitations of the approach were discussed, particularly the lack of therapist presence during therapy delivery:

Instilling the hope, the computer cannot instill hope.

Health care professional, male

Furthermore, they felt that computerized therapy delivery was not suitable for all, especially in high-risk situations, and expressed concern about what would happen to young people if they felt particularly low following completion of computerized treatment sessions.

#### Essential Elements Required for a Successful BA Program

Goal setting, activity scheduling, coping skills, mood or activity recording, and monitoring feelings were deemed the most important criteria for inclusion in the new program. Although goal setting was regarded as a *vital* component in assisting therapists to gauge treatment direction, health care professionals also felt it was important for young people to monitor their mood regularly and identify factors affecting it. Most users also wanted to see information about depression, problem solving, and quizzes included. However, there was concern about how components such as goal setting and problem solving could be implemented in a computerized format, given that they are person-centered and individualized approaches. Some users wanted homework to be included, but they stipulated that this needed to be presented under a different name and monitored with reminders sent to support engagement.

To increase inclusivity, health care professionals suggested presenting information in different modes and allowing users to adjust program settings to enhance ease of use. They also wanted users to be able to ask questions about anything they did not understand and have key points revisited in each session to assist understanding.

#### Developmental Considerations and Flexibility of the Program

It was felt that more than one presentation of the new program, based on developmental level, was needed. There was concern that some young people would not understand particular words and others, especially those with low mood or depression and lacking energy, would struggle if presented with a lot of text. Therefore, breaking words down to aid understanding, using combinations of words and pictures in the program, and embedding an audio option so that content could be read aloud were suggested.

The health care professionals wanted both session frequency and length to be tailored to each user with consideration given to energy levels, depression severity, developmental level, and the number of questions a young person had. One individual, highlighting that those with low moods might have shorter attention spans, proposed basing sessions upon graded exposure. Overall, the consensus was that approximately 8-12 sessions, delivered weekly and lasting between 30 and 45 minutes, would be optimal.

#### Depression Severity and Care Pathway Positioning

It was felt that those with severe low mood or depression might have difficulties using a computerized program; therefore, it would be most suited to those experiencing mild-to-moderate low mood or depression. Aligning to this, tiers 2 (early help and targeted services) and 3 (specialized services) of CAMHS and step 2 of the children and young people’s improving access to psychological therapy stepped care model (low-intensity services) were suggested as areas where the new program would be best placed.

Health care professionals felt that the new program could be positioned anywhere in the care pathway. First, it could be used by young people awaiting treatment, thus reducing the number of subsequent face-to-face appointments and allowing more time to be allocated to those with more severe depression. Another recommendation was for the program to be used alongside usual care, either between or during face-to-face sessions with the therapist present. Particular benefits of this included providing young people with a new medium within their care and providing therapists with an additional tool to monitor progress. Finally, it was proposed that the new program could be offered as a *further treatment* and used before discharge from CAMHS.

#### Risk Monitoring

Several recommendations were made about how to identify anyone deemed at risk following computerized therapy and how to manage this situation. These included incorporating live chat or crisis numbers into the new program, allowing young people to request a call back from a health care professional if they were feeling at risk and encouraging users to take control and agree to seek additional support if required. To implement this, one health care professional suggested using contracts:

You could have it in the contract...so just something that they signed to say that “if you do feel at risk or you feel that you need to talk to someone that you are willing to contact the crisis team and speak to someone,” even the Samaritans.

Health care professional, female

#### Systemic Approach and Collaborative Working

Therapeutic alliance was particularly important to health care professionals. They felt that during the treatment process, clinicians were required to instill hope and monitor both progress and deterioration with individual clients, providing support accordingly. They were therefore concerned about the lack of human contact inherent to computerized therapy delivery and, although supportive of the new program, wanted treatment delivery to incorporate a balance between the computer and therapist:

I think for me though there is nothing more powerful that the connection between the therapist and the client, you know.

Health care professional, female

Health care professionals felt that parental involvement was also essential, particularly regarding risk monitoring, and reported that therapies were often more successful if parents were included. However, they acknowledged that relationships with parents can be difficult, and therefore, parental support within the new program would need to be adapted based on user-parent relationships.

Finally, it was highlighted that, for treatment success, a systemic approach to therapy delivery was required with the cooperation of multiple agencies. They contended that everyone had a role during therapy delivery, and therefore, it was important to be flexible and ensure roles were clearly defined:

Everybody has a role to play from the preventative early intervention, the mild end, the moderate end, the severe end so it will be quite clear who does what, when, and how, and then that maxes our public money, so we’re not stop starting.

Health care professional, male

Through collaboration, it was felt that multilevel monitoring would be in place throughout treatment completion, with young people having a variety of support contacts when required, something especially important if the risk was presumed.

## Discussion

### Principal Findings

This study allowed vital information to be collected from various perspectives to inform the development of a new computerized BA program for young people. Previous research suggests that differences exist between the views of young people and health care professionals [27,28], with the limited effectiveness of some programs attributed to a failure to tailor interventions to patients and practices [19,20]. Therefore, the views of both young people and health care professionals were collected and incorporated into the development of the new intervention.

The findings from this qualitative work demonstrated that both young people and health care professionals endorsed the use of computerized therapy delivery, and the advantages of the approach were identified. These included enhanced anonymity, treatment accessibility, and awareness; these findings were concordant with previous research in the area [15,16,18]. Through the focus group and interview discussions and the associated activities completed, the contention that BA may be an acceptable treatment for delivery in this context was supported, as was support for future clinical effectiveness research. When presented with potential content for inclusion in the new intervention, based on techniques commonly delivered within BA, all content was selected by at least one-third of participants in each group. Furthermore, two of the main approaches consistently delivered within the BA, activity scheduling and activity monitoring [30], were selected for inclusion by most participants.

Apart from providing support for the development of a new computerized therapy program based on BA, this qualitative work also provided vital information regarding the presentation, delivery, and content of the new program to ensure that it meets the needs of the target user.

Although there were individual differences across the 3 participant groups, with novel themes emerging from each (Figure 1), agreements were evident about several issues pertaining to the presentation and delivery of the program, which were used to inform its development. For example, all groups felt that more than one presentation was required to suit the target age range, and it was generally felt that program sessions should last approximately 30-45 minutes with appropriate time in between sessions, and all groups wanted the program to be easily accessible.

However, opinions regarding some of the program content were more complex to reconcile, both in incorporating the views of the 3 participant groups where differences were apparent and also being mindful of the need to include evidence-based components of therapy.

Across all groups, concern was expressed about the inclusion of homework, with this component regarded as less important than others. Despite these concerns, research has demonstrated correlations between homework compliance and clinical improvements when delivered within CBT [34]. Including homework in the new program was therefore important, despite being unpopular. To ensure a balance between incorporating evidence-based components within therapy but being sensitive to the opinions of the target users, both were considered. Although they would generally have preferred homework to be omitted from the new program, the participants stipulated that, if included, it required a different name and had to be minimal. Therefore, in the development of the new program, these stipulations guided the inclusion of a homework component.

Discussions also occurred regarding the involvement of others in the completion of a computerized therapy, particularly parents. As reported elsewhere in a systematic review of young people’s experiences of using technology-assisted CBT [17], both positive and negative opinions regarding parental involvement were expressed. Although the community sample would have preferred no involvement from others, they agreed that in instances of risk, parents needed to be notified; the service users were generally more positive and identified how treatment effectiveness could be enhanced if parents were involved. In contrast, health care professionals regarded the involvement of others, both parents and health care professionals, as essential, contending that both needed to take an active role in the new program. Research has highlighted that the need for autonomy among young people can be a barrier to seeking mental health support [35]. Therefore, this clearly needs to be addressed in the development of the new program while considering the important perspectives captured within this study. Researchers argue that interventions need to address the autonomous needs of young people to increase their likelihood of seeking help and suggest helping young people to know how and when to seek support from others [35]. Therefore, in the new program, development focus needed to be placed upon supporting help-seeking while remaining sensitive to young people’s need for autonomy.

The needs of young people can be better met if there is knowledge about where they seek help when they are distressed [36]. Despite different experiences, the young-person participants expressed concerns related to accessing support, particularly concerning information disclosure and how they would be treated and perceived as a result. They felt that a computerized therapy would allay some of these concerns while allowing them to access therapy at times when most needed and in their own surroundings where they feel most comfortable. Similar findings have been reported in previous research [37,38], which highlights the benefits of adopting a computerized therapy delivery approach in ameliorating some of the barriers faced by young people in accessing services. However, although the health care professionals in this study were familiar with the availability of computerized therapies, few young people were, as none were directed to any resources in the past. This is represented in Figure 1, which shows that although familiarity with computerized therapies emerged as the main theme for health care professionals, this was not the case for young-person participants who instead discussed the requirement of promoting awareness. This demonstrates the need to sufficiently promote the support available to young people.

Similar to previous research [27,28] and as presented in Figure 1, the differences in the experiences, opinions, and priorities across the participant groups in this work highlight the need to incorporate different stakeholders in the development of new therapies. Although it is clear that the opinions of young people need to be considered within their own care, the views of health care professionals also need acknowledgment, as those who participated in this study provided salient information from a clinical perspective. Furthermore, the opinions of those who do not routinely access services but for whom a treatment may be useful need to be considered in therapy design. All young-person participants were from similar age ranges and ethnic groups and lived within relative geographical proximity. Despite this, clear differences in opinion were evident based on whether they had previously accessed support. As discussed, 65% of young people with low mood or depression do not access care [1] because of factors including stigma [3,4], negative attitudes about help-seeking [4], accessibility [3], and reluctance to engage one-to-one with a therapist [5]. Thus, although it is undoubtedly important to tailor treatments to target users [39], the views of those with limited experience of CAMHS also need to be considered.

Therefore, a systemic approach to the development of new interventions needs to be adopted to ensure that they meet the needs of the target user. Such an approach should incorporate the views of those with treatment experience, who are experts on what works and what does not; those who have not accessed treatment, who are the experts on the barriers and how these may be overcome; and health care professionals, who are experts on the practical application of therapy and its integration into services.

### Limitations

Owing to several limitations of this study, the results need to be interpreted with caution. To ensure that rich and trustworthy information was collected as part of this study, 3 different participant groups were recruited. Despite this, several factors may have affected the transferability of the findings. As an opt-in method was used, the young-person participants were more likely to be those motivated to engage and thus might not have expressed views demonstrative of the general age range population. Only one service user participant was male, which, although unsurprising with research suggesting that males are less likely to access mental health services [40], meant that the views of males were underrepresented within this sample. Furthermore, although recruitment occurred within 4 NHS trusts, these were all located in close proximity to each other and were culturally similar, with little ethnic diversity in the overall sample. Therefore, the views expressed might not be typical of health care professionals based within the NHS trusts or young people receiving support from other NHS trusts.

### Conclusions

Vital information was collected to inform the development of a new computerized therapy. Although similarities in the opinions of the participant groups were evident, differences highlighted the need to adopt a systemic approach in therapy development. In the context of young-person therapy, the opinions of young people with and without experience in accessing mental health support and health care professionals need to be incorporated.

## Appendix

Multimedia Appendix 1 Young person participant and health care professional interview schedules.

## Abbreviations

BA: behavioral activation
CAMHS: child and adolescent mental health services
CBT: cognitive behavioral therapy
GP: general practice
NHS: National Health Service
