# Supplementary material for: Perceptions of and Opinions on a Computerized Behavioral Activation Program for the Treatment of Depression in Young People: Thematic Analysis
Source: J Med Internet Res. 2021 Apr 13;23(4):e19743. doi: 10.2196/19743 (PMC8080144; doi:10.2196/19743)
Supplement: Multimedia Appendix 1 [file jmir_v23i4e19743_app1.doc]

#


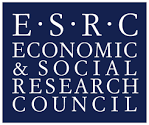


**FOCUS GROUP/INTERVIEW SCHEDULE: Young people**

**Previous experience of accessing online help**

1. What online help have you ever accessed?

*If yes:*

1. Can you tell me a little about the types of help you have accessed?
2. Did you find online help useful?
3. Was there anything about the online help that you didn’t like?

*If no:*

1. Are there any reasons why you haven’t accessed online help? *[prompts: accessibility, not interested, unaware it existed, etc.]*

**Programme development (views and acceptability)**

1. In what ways do you think young people of your age (between 11 and 16) would benefit from using a computerised programme like the one we are looking to make?
2. What should our computerised programme be called?

**Programme components/activities**

1. If you were to use a computer programme like the one we are planning to make what sorts of activities would you like to see on it? *[prompts: information? Quizzes? Homework? Rewards (i.e. certificates)]*
2. Should the programme also have handouts or information sheets that can be printed off?

**Suggestions for treatment-related activities/goals/organising time**

[**Activity 1** – young people/person will be presented with a chart showing the different types of activities that could be included in the programme and asked to place stars next to the things they think should be included]

1. Was there anything that you think was missing from the list that should be in the programme?

**Length and number of sessions**

1. How many sessions do you think the programme should have?
2. How long do you think each session should last?

**Presentation (Colour, interactive)**

[**Activity 2** - Young people/ person will be given a list showing a number of factors relating to the programme and asked to rate them in terms of importance as to what they think would be most important to make the programme attractive to them]

**Location (Schools, home, CAMHS)**

1. Should the computerised programme be available anywhere (i.e. online) or should it only be available in places like schools, doctor’s surgeries etc.?

**Follow up sessions**

1. Do you think there should be additional sessions to be completed after the programme has finished (like follow up sessions, reminders of what you have learnt) or all sessions completed together?

**Different versions for different ages**

1. Do you think one programme would be suitable for young people aged 11 to 16 or do you think we would need to make a number of programmes for different ages?

*If different versions*

1. What ages should the programmes be designed for?

**Parental involvement**

1. If you were to complete a computer programme like this would you like to have your parents involved too *[prompts: parental sessions, sessions to be completed together]*

*If yes:*

1. How would you like them to be involved?

*If no:*

1. Why would you not like your parents to be involved?

**Dissemination**

1. When the computer programme has been made what is the best way for us to let young people know about it and encourage them to use it if they are feeling low/depressed?

**FOCUS GROUP/INTERVIEW SCHEDULE: Healthcare Professionals**

**Awareness of any similar programmes that are currently available**

1. Have you heard of or used behavioural activation or activity scheduling in clinical practice?
2. To what other online or other sources of help do you ever refer young people experiencing depression to?

**Components to be included in a programme**

[**Activity 1 –** Healthcare professionals will be presented with a list of different types of activities that could be included in the programme and asked to select the things they think should be included]

1. What was missing from the list that should be in the programme?

**Target audience**

1. Who do you think the proposed programme would work best with? [*prompts: age range, severity of depression*]

**Length and number of sessions**

Based on your therapeutic experience:

5. How many sessions do you think the proposed programme should contain?

6. How long should sessions last?

7. How frequently should sessions be completed? [*prompts: monthly, weekly, bi-weekly*]

**Location (Schools, home, GP surgeries)**

8. Should the computerised programme be available anywhere (i.e. online) or should it only be available in places like schools, doctor’s surgeries etc.?

**Position: a programme could be placed within the current care pathway**

9. How and where would the proposed programme fit within the current care pathway?

**Supporting completion of a programme**

10. Who do you think should co-ordinate use of a computerised programme?
